# Novel Framework for Quality Control in Vibration Monitoring of CNC Machining

**DOI:** 10.3390/s24010307

**Published:** 2024-01-04

**Authors:** Georgia Apostolou, Myrsini Ntemi, Spyridon Paraschos, Ilias Gialampoukidis, Angelo Rizzi, Stefanos Vrochidis, Ioannis Kompatsiaris

**Affiliations:** 1 Information Technologies Institute (ΙΤΙ), Centre for Research and Technology Hellas (CERTH), 57001 Thessaloniki, Greece; nmyrsini@iti.gr (M.N.); sparaschos@iti.gr (S.P.); heliasgj@iti.gr (I.G.); stefanos@iti.gr (S.V.); ikom@iti.gr (I.K.); 2 FIDIA S.P.A., 10099 Torino, Italy; a.rizzi@fidia.it

**Keywords:** Industry 4.0, CNC machining, metal cutting, vibrations, condition monitoring, quality control, artificial intelligence

## Abstract

Vibrations are a common issue in the machining and metal-cutting sector, in which the spindle vibration is primarily responsible for the poor surface quality of workpieces. The consequences range from the need to manually finish the metal surfaces, resulting in time-consuming and costly operations, to high scrap rates, with the corresponding waste of time and resources. The main problem of conventional solutions is that they address the suppression of machine vibrations separately from the quality control process. In this novel proposed framework, we combine advanced vibration-monitoring methods with the AI-driven prediction of the quality indicators to address this problem, increasing the quality, productivity, and efficiency of the process. The evaluation shows that the number of rejected parts, time devoted to reworking and manual finishing, and costs are reduced considerably. The framework adopts a generalized methodology to tackle the condition monitoring and quality control processes. This allows for a broader adaptation of the solutions in different CNC machines with unique setups and configurations, a challenge that other data-driven approaches in the literature have found difficult to overcome.

## 1. Introduction

Standards in modern manufacturing have been significantly raised over the past years. Industries are continuously evolving their processes in order to cover user needs by increasing their production, eliminating operating costs, possible errors, and defects, and ensuring the final-product quality. CNC (Computer Numerical Control) machines are broadly used by numerous industries for high-precision machining; these are tools that convert a raw metal workpiece into a finished, complete product by using drilling, milling, and turning operations to remove the extra material and give the final form to the product [1]. Following the standards and requirements of Industry 4.0, CNC machines have started using advanced control technologies, such as artificial intelligence (AI) systems and machine-learning mechanisms [2,3].

This integration of advanced technologies has ushered in a new era of CNC machining, one characterized by unprecedented capabilities and transformative potential. Industry 4.0 marks the confluence of digitalization, automation, data-driven decision making, and connectivity, all orchestrated to enhance efficiency and drive innovation. CNC machines, already celebrated for their high-precision and high-quality machining abilities, have seized upon this wave of technological progress.

Many articles on CNC machining, artificial intelligence (AI), and machine learning (ML) have been published over the past years, from state-of-the-art reviews to articles on recent developments and real-life industrial applications [4]. The interest of the scientific community has turned towards this direction because these machines have evolved into intelligent entities that are capable of autonomous decision making, real-time monitoring, and adaptive adjustments. They are no longer just tools but sophisticated partners in the manufacturing process, equipped to optimize operations, reduce waste, and ensure consistent quality. As CNC machines become increasingly interconnected within the broader manufacturing ecosystem, they contribute to the creation of “smart factories”, where data flows seamlessly from machine to machine, and insights derived from AI and ML algorithms are used to fine-tune processes in real time. This connectivity fosters agility, enabling a rapid response to market dynamics and customer demands [5].

Moreover, the parameters that influence the quality of the final product that is produced by the CNC machine have also been examined in many works. In the realm of CNC machining, achieving exceptional product quality extends beyond the mere precision of tool movements. It encompasses a wide array of factors, ranging from the tool selection and cutting speeds to tool-path strategies and the workpiece material properties. Researchers have delved into the minutiae of each of these parameters, dissecting their impact on the final product with meticulous precision. Studies have explored the optimization of the cutting parameters, seeking the ideal balance between the feed rates and spindle speeds to minimize tool wear and maximize the material removal rates. The choice of cutting tools, with their varying geometries and materials, can significantly influence the surface finish and dimensional accuracy. Additionally, the workpiece characteristics, such as the hardness and thermal conductivity, pose unique challenges that require tailored machining approaches for optimal results. Furthermore, investigations into tool-path-planning and optimization strategies have revealed that the sequence in which the CNC machine processes a workpiece can profoundly affect both the efficiency and quality. Researchers have developed algorithms and methodologies to generate tool paths that minimize vibrations, reduce tool deflection, and optimize chip evacuation, ultimately contributing to the enhancement of the product quality. An interesting review was published by Ntemi et al. [6] in which all the studies on CNC machining were collected to find the non-directly inferred related gaps among them.

Therefore, it is necessary to use advanced ML in order to monitor the machines and ensure the high quality of the products. These techniques leverage real-time data and algorithms to dynamically adjust the machining parameters, compensating for variations in the material properties, tool wear, and environmental conditions. The result is a harmonized, adaptive machining process that maintains exceptional product quality even in the face of external disturbances. For this purpose, infrastructure monitoring (IM) and rapid quality diagnosis (QD) tools for machine condition monitoring and product quality monitoring are used, respectively, serving as indispensable components in CNC machining. Machine condition monitoring aims to monitor and track the condition of the machine in real time in order to identify possible errors or malfunctions and, if necessary, take actions to prevent the errors or even correct them [7]. Product quality monitoring aims to offer intelligent techniques that will improve the quality of the final product [8]. In this era of smart manufacturing, the CNC machine’s role extends beyond precision; it is an enabler of agility and competitiveness.

The main contributions of the AI-powered data-driven approaches that have been adopted by the CNC manufacturing industry are summarized as follows:

- Ensuring the final surface quality: The estimation of the final surface quality is performed by correlating the processing conditions (axial and radial depths of cut, axis feeds, spindle rotation speed, workpiece material, tool geometry) with the final workpiece quality, which requires an artificial intelligence (AI)-based tool that is capable of extending over multiple dimensions. Appropriate testing sessions allow for training the algorithm with the provision of quality measurement, performed after the process ends with a Mitutoyo surface roughness tester;
- Chatter detection: A fast Fourier transformation (FFT) analysis of the vibrations and their correlation with the current processing conditions (spindle speed rotation, number of tool flutes) allows for determining whether chatter is taking place during the process. An empirical stability diagram is built during a testing campaign to support the implementation of an AI-based algorithm for the online-chatter-presence removal (through specific feed and spindle speed override signals);
- Evaluation of the machine tool condition: Big Data solutions are required to analyze the trends of the processing signals, such as the positions, speeds, currents, and torques, collected during the periodical execution of dedicated reference tests. Variations in these parameters during otherwise identical tests allows for identifying degradation patterns (compared to nominal conditions) via an unsupervised-machine-learning (ML) approach, as well as possible faulty components (failure modes). New processing constraints are imposed on the equipment to reduce the impact of the failures on the process while waiting for maintenance intervention.

## 2. Related Work

In this section, we provide an overview of the existing research and developments in the field of vibration monitoring for CNC machines. Vibration monitoring plays a pivotal role in ensuring the accuracy, reliability, and efficiency of CNC machining processes. Chatter affects the product quality significantly [9,10]. For this reason, a plethora of works have emerged over the past decade studying vibrations [11].

Many monitoring tools rely on sensor signals. For example, Ref. [12] utilized a wireless sensor network tool to improve the efficacy of the data acquisition within the context of a real-time, automated monitoring platform developed for smart manufacturing. The wireless sensor networks have been proven to be very efficient, as they are cost-effective, easily installed, and provide physical mobility when it is required. Smart infrastructure monitoring is not only restricted to the acquisition and tracking of sensor signals that are installed in industrial environments. It is also responsible for estimating the status of the production lines and processes through the analysis of the sensor signals, and for providing proactive alerts if a harmful event is estimated to be present [13]. Timely and highly accurate problem detection may ensure a minimum downtime in the production line. ML has been widely used for this purpose. ML for smart infrastructure monitoring involves process monitoring, problem detection, quality control, process status diagnosis, machine status monitoring (e.g., health), and predictive maintenance [14].

The manufacturing industry’s CNC machining processes are a prime example of an advanced infrastructure-monitoring application. Due to component deterioration and subpar machine tool parameters, the integration of monitoring techniques into these production lines has been shown to be incredibly successful in predicting impending machine failures and product defects. As a result, expensive machinery failures and production line stops can be avoided, enabling a manufacturing process that is more effective. Tool wear or component degradation in CNC cutting processes is caused by the cutting tool’s constant contact and relative sliding with the workpiece. Friction dynamics produce this phenomenon, which has a significant impact on the final surface quality of products. This is the reason why machine-learning researchers all over the world are interested in tool wear prediction. Tool wear is typically the dependent variable, and the feed rate, cutting time, spindle speed, and cutting depth are the independent variables. Through the use of a multi-sensor tool wear prediction method based on stationary subspace analysis (SSA), support vector machines (SVMs) have been successfully applied for tool wear prediction, tool status identification, and fault diagnosis [15,16,17]. Without any prior knowledge, multi-sensor signals are converted to stationary and non-stationary sources. Tool wear estimation for small data samples is performed using least squares (LS) and support vector regression (LS-SVR). The incorporation of SSA significantly improved the LS-SVR tool wear prediction performance, despite the limited data sample size. Because of their capacity to (1) infer statistical relationships between the tool wear condition and observed sensor signals, and (2) model the transition between different tool conditions, hidden Markov models (HMMs) have been widely applied for tool wear monitoring [18]. In [19], an enhanced online HMM is suggested to calculate the tool wear state and forecast the RULs of cutting tools, taking into account switching the cutting conditions. Artificial and deep neural networks (ANNs, DNNs) have been used extensively in tool wear detection recently due to their exceptional performance in handling large volumes of data and solving nonlinear problems [20,21,22]. Tool wear has also been detected using convolutional neural networks (CNNs) on machined surface images [23,24]. Convolution and pooling processes exhibit high detection accuracies because they can describe the internal relationship from the input feature maps that are taken from machining images. Nevertheless, they necessitate the costly installation of cameras on the CNC machinery. Recurrent neural networks (RNNs) have been extensively used for tool wear monitoring over the last ten years because of their exceptional ability to capture long-term dependencies and track time-evolving entities closely [25,26,27,28]. For example, in [29], online one-timestep-ahead and two-timestep-ahead tool wear prediction is performed by combining a Gated RNN (GRNN) with an LSTM NN. In other words, using past tool wear data, the objective is to forecast the tool wear at a given timestep or at a later one. The fact that this method can be applied to various machine tools and scenarios is one of its main advantages.

Because tool wear prediction depends on processing sensor signals, it is closely related to time-series and signal-processing analyses. Because particle filters (PFs) can model the nonlinear and non-Gaussian dynamics governing the sensor time series, they have been used in several works to predict tool wear. The idea behind PFs is that, by using the Monte Carlo method, a set number of particles, or samples, can represent any kind of probability density function. In [30], a Fusion-PF (FPF) was used to apply fusion on simulated and sensor data in order to closely monitor the evolution of the tool wear over time. Applying the USUI tool wear model to values obtained through finite element model (FEM) simulation, in which the wear rate is a function of the absolute temperature, relative velocity, and constant pressure at the contact surface, yields cutting simulations. The evolution of tool wear is controlled by dynamic characteristics that vary over time and are nonlinear. To account for this, a PF in [31] models the nonlinear dynamics of the tool wear evolution over time using Bayesian inference. This model is then incorporated into an LSTM NN to forecast the in-process stochastic tool wear progression using past data. According to the PF, the degradation trend equation is the state transition equation. To infer the instantaneous uncut chip thickness, nonlinearities resulting from tool run-out and trochoidal cutting-edge trajectories are combined with the in-process stochastic tool wear values. The input to the PF-LSTM is formed by the translated in-process tool wear data and the measured cutting forces and tool vibration signals. Experiments have demonstrated that by accounting for the influence of the stochastic tool wear, the prediction accuracy can be significantly increased, and the useful life of the cutting tool can be extended by 19%.

Fuzzy logic theory uses multi-valued variables to describe the partial correctness and partial wrongness of a state, instead of using the bi-valued variables (such as 0 and 1) suggested by classical logic. This approach eliminates the need for explicit mathematical modeling. As a result, numerous fuzzy techniques have been put forth to identify tool wear [32,33]. Tool wear detection is one area in which the adaptive neuro-fuzzy inference system (ANFIS) has been extensively used [34]. Takagi–Sugeno fuzzy inference is the foundation of the adaptive multi-layer feed-forward network technique known as the ANFIS. It maps inputs onto an output by utilizing fuzzy reasoning and NN schemes. It models nonlinear functions, predicts chaotic time-series evolution, and identifies the on-line nonlinear components of a system by combining gradient descent, least-squares, and back-propagation algorithms [35]. The ANFIS is used extensively for chatter detection and is taught by the vibration and communication particle swarm optimization algorithm (ANFIS-VCPSO) in [36], allowing for real-time tool wear monitoring. To determine the ideal set of milling parameters, the VCPSO algorithm evaluates the multi-objective optimization based on the minimum cutting power, surface roughness, and maximum material removal rate. Tests have demonstrated that ANFIS-VCPSO performs better than the most advanced techniques, and that the newly introduced VCPSO algorithm has the capability of global optimization.

QD is a fundamental component of industrial intelligence, in which the product quality is evaluated, and critical feedback is produced to suggest corrective actions and improve the product quality [37]. To ensure highly accurate product quality estimations, intelligence should be utilized in inference systems. Such intelligent systems extract latent complex insights about the product quality from sensor signals and provide proactive warnings or alerts about imminent defects [38]. Several ML methods have been proposed over the past years for product quality estimations [39,40].

The two main focuses of the CNC-related industrial segment are surface roughness prediction and chatter detection. Chatter, which is the vibration produced during the machining process, is one of the most significant underlying reasons for poor product quality. It is possible for the workpiece to move slightly while the machine tool is operating, which can result in non-resonant vibrations. Chatter is associated with self-excited vibrations produced by the regenerative effect in the tool or workpiece. The effect mentioned above is brought about by a periodic modulation of the uncut chip thickness that is incorporated into the structure’s eigenfrequencies [41]. It is a self-excited-vibration phenomenon brought on by friction created between the workpiece and the tool, thermomechanical effects, and the relative motions of the machine’s parts. Because of the internal or external movement of the machine’s structure, the vibrations cause disruptions to the machine’s normal operation. This can result in low productivity and faulty parts, which can damage the machine over time. Consequently, the research community has become interested in chatter and is working to identify, forecast, and eradicate it. The tool operators need to properly regulate the cutting parameters, like the rotational speed and cut depth, to prevent chatter. Stability lobe diagrams (SLDs) are used to track the stability of the cutting parameters and can help operators to identify the best set of parameters to reduce chatter; however, analyzing them is a time-consuming and difficult task [42]. PCA has been widely used in numerous smart manufacturing monitoring approaches and has been mentioned as a useful substitute for other feature selection techniques [43,44]. Another suggestion for chatter identification is made in [45], where the detection technique is predicated on using logistic regression (LR) to classify the status. In [46], after performing a spectrum analysis on the acceleration signals gathered from a CNC milling system, an HMM-based chatter classification model was used. Because deep-learning approaches to monitoring, and ANNs in particular, perform better than statistical approaches, they are becoming increasingly common in chatter detection systems. A deep multi-layer perceptron (DMLP)-based chatter detection framework is presented in [47]. This framework is capable of formulating nonlinear solutions, which leads to a more accurate capturing of the relationship between the cutting parameters and chatter presence estimations.

Numerous studies have been conducted on the topic of efficiently detecting and predicting chatter in industrial cutting machinery by combining statistical learning techniques with signal processing. In [48], the effectiveness of various statistical classifiers was compared in a chatter detection scenario with signal-processing methods, like ensemble empirical mode decomposition and wavelet packet transform. An intelligent chatter detection method with a similar pipeline-based diagnosis strategy is proposed in [49]. In order to identify chatter, the system first collects three-axis vibration signals from the accelerometers. Then, it uses signal processing, feature extraction and selection, and classification. In addition, the authors of [50] examine the idea of dimensionality reduction in chatter diagnosis and state change detection in machine processes. In [51], a different deep-learning method for chatter detection is put forward. Its basic idea is to monitor online chatter using a self-organizing map (SOM) neural network. When it came to chatter detection, fast Fourier transform (FFT) was unquestionably the gold standard in signal processing for a very long period [52,53]. The FFT enables the shift to a frequency domain where identifying the resonant chatter frequencies is simpler. Local peaks that indicate the existence of chatter can be used to identify the dominating frequency bands in the FFT spectrum analysis of the vibration signals. Chatter is presumed to be present if the high-energy vibration frequencies are near the milling machine’s inherent frequencies. The feature extraction potential of variational mode decomposition (VMD) has also been examined in a number of studies. When the energy distribution in the frequency spectrum is modeled in conjunction with VMD, chatter may be effectively detected. A variant of VMD known as optimized VMD (OVMD) is suggested in [54,55] in conjunction with multi-scale permutation entropy (MPE) to detect noise in milling operations. VMD is also employed in [56], in which the kurtosis of the vibration signals is used to automatically determine the settings. A kurtosis rise results from the signal’s distribution departing from its steady state when chatter occurs. Fuzzy-logic-modeling methodologies are chosen when the underlying system’s complexity and ambiguity make it impossible to precisely simulate the milling process. Fuzzy logic techniques have gained a lot of traction in chatter detection because of their ease of use and ability to handle nonlinear situations [57]. In order to predict the presence of chatter, a CNC cutting machine’s acoustic signals were collected and the ANFIS was applied in [58]. The ANFIS was also utilized in [59] to investigate the relationship between the cutting parameters and the intensity of chatter. Resonant vibrations cause the spindle and other mechanical components that regulate the machine’s motion to move when the generated vibrations approach the machine’s natural frequency. Such erratic movements, improper cutting parameters, and even tool wear [60] can leave grooves on the surface of the workpiece, which lowers the quality of the final product. Surface roughness is therefore a direct indicator of the quality of a machined product, and any deviation from the nominal surface form results in increased production costs because of the need to rework or discard failed parts.

A major limitation of the models described here is the inability to generalize them (i.e., the inability to apply them in real machining production processes). Specifically, the models identified in the literature exhibit limitations when faced with changes in the machining conditions, as they are often trained on specific parameter ranges and fixed labeled datasets. The absence of annotated data in real-life industrial conditions further complicates the model inference, necessitating the estimation of tolerance parameters for the dependent variable. A plethora of experiments and real-life application scenarios have shown that both the IM and QD tools confront this issue, as they have been used successfully in different use cases and datasets through the exploitation of the vibration data, which are prevalent in CNC machining.

## 3. Methodology

We address the mentioned problem by combining advanced vibration-monitoring methods with the AI-driven prediction of the quality indicators. First, a novel add-on kit to monitor the behavior of the cutting process was developed and integrated. This kit includes a smart flange that integrates accelerometer and temperature sensors working very close to the source of the vibration. These signals feed AI-driven (machine learning), trained algorithms that predict (in-process) the expected quality (e.g., in terms of the surface roughness) of the machined workpieces. Based on the predicted quality, the AI algorithms recommend the best configuration of the CNC adaptive controller’s process parameters in the case that some drift/deviation is detected. The process is complemented, at least in the training phase, with data coming from the inspection of already-machined workpieces to refine the AI models and algorithms. As a result, the solution reacts in advance to deviations that could cause undesired defects on the surface, producing workpieces with smoother surfaces. The solution aims to increase the quality, productivity, and efficiency of the process, as the number of rejected parts, time devoted to reworking and manual finishing, and costs are reduced considerably.

### 3.1. Infrastructure Monitoring (IM)

**Figure 1:**
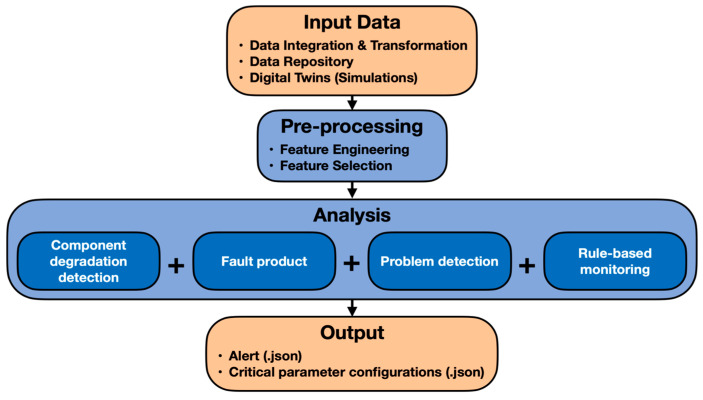
Conceptual architecture of machine-condition-monitoring IM tool.

The goal of infrastructure monitoring (IM) is to efficiently notify users when an issue is identified by combining a variety of monitoring tools with predictive failure-alerting mechanisms. It tracks and analyzes industrial sensor signals to keep an eye on the production lines and processes. In order to offer input regarding the best possible operation of the machines, it also keeps track of the real-time analysis results that are provided by other solutions. To identify or foresee impending issues in the production lines, it uses machine-learning algorithms. In addition to the proactive alerts, it offers the parameter configurations of the observations for which an estimated failure is present. This could help machine operators with any necessary decision making when it comes to reconfiguring the parameters to avoid irreversible machine failures. Figure 1 shows the conceptual framework of IM.

#### 3.1.1. IM Framework for Component Degradation Detection

The strategic steps that were taken to design the machine-condition-monitoring tool that comprises a fully functional component degradation detection framework, which can be generalized and easily used in different monitoring use cases (and the respective datasets), are the following:

1. Find a well-established public component degradation dataset similar to that of the user’s;
2. Select a set of classifiers that have already been successfully applied on a similar problem, according to the literature;
3. Conduct experiments using the public dataset and evaluate the detection performance of the classifiers;
4. Apply feature engineering on the user’s dataset;
5. Choose the classifier that performed the best;
6. Apply the best-performing classifier to these datasets;
7. Fine-tune/optimize the algorithm to achieve optimal detection accuracy;
8. Design/develop the final output of the solution;
9. Create the user interface.

#### 3.1.2. Feature Engineering

Feature engineering was applied to enhance the classification efficiency. The total number of samples comprising the dataset is 104. The reason for such a small sample size is the fact that different numbers of entries were recorded for each feature. Consequently, concatenation was not applicable. There are two possible solutions to this problem: (1) interpolate the feature values and produce all the possible combinations as extra samples, or (2) compute the average value of the feature values. The second solution was applied in order to ensure computational feasibility. As a result, the number of samples is equal to the number of experiments. Five-fold cross-validation was applied to construct a training set including 72 samples (70%) and a test set including 32 samples (30%). Furthermore, except for the average value of each feature entry, the respective median value was computed to introduce an extra feature enhancing the performance of the LGBM. Last, the fast Fourier transform (FFT) of the position of the Y-axis was also introduced to enhance the tool wear detection performance. The results are discussed next.

#### 3.1.3. Choosing the Optimal Classification Approach

Because the objective is to estimate whether a milling tool is either worn or unworn, binary classifiers have been utilized. For the experimental procedure, the support vector machine (SVM), random forest (RF), extreme gradient boosting machine (XGBoost), and light gradient boosting machine (LGBM) classifiers were implemented and evaluated:

- SVM: The objective of SVM is to find a hyperplane (or decision boundaries) in a D-dimensional space (where D denotes the number of features) that provides the maximum separability of the data points. That is, the ultimate objective is to find the optimal hyperplane that has the maximum margin, ensuring the maximum distance between the data points of two different classes, which, in our case, are degradation/no degradation. To maximize the distance between the data points and hyperplane, a hinge loss function is utilized. Next, the partial derivatives of the objective function with respect to the weights are calculated to compute the gradients and update the weights;
- RF: The concept idea in decision trees is to predict the value of the dependent variable by learning the decision rules based on the data features. RFs are ensemble-learning methods, which utilize multiple learning approaches to provide an optimal estimation performance. At each training timestep, it constructs a multitude of decision trees. The final decision regarding the classification of an observation (RF output) relies on the class selected by the majority of trees;
- XGBoost: The building block of this classifier is the gradient boosting machine algorithm, which utilizes decision trees. The boosting technique is an ensemble method that sequentially adds models to the ensemble. Here, the decision trees are added one by one. The latter trees fit and correct the performances of the prior ones. The gradient descent optimization algorithm and arbitrary differentiable loss functions are utilized to fit each tree. The loss gradient is minimized when the model fits well;
- LGBM: This classifier is also a gradient-learning framework based on both the boosting technique and decision trees. In contrast to the XGBoost, the LGBM utilizes histogram-based algorithms to reduce the memory consumption and speed up the training process, while adopting a leaf-wise growth strategy with depth constraints. The histogram algorithm classifies floating-point eigenvalues into n number of bins and constructs an n-th width histogram. This partitioning reduces the amount of required memory and increases the execution speed, while the accuracy of the model is not affected. Furthermore, the leaf-wise strategy for the growth of the decision trees is much more efficient than the level-wise one used by the XGBoost. The leaves with the highest branching gains are selected each time. In this way, more errors are reduced, and the accuracy is significantly enhanced. Last, the LGMB allows for a plethora of parameter tuning that can ensure overfitting elimination.

Experiments have shown that, out of the classifiers described in Section 3.1.3, the best-performing classifier for the tool wear detection use case is the LGBM. More experimental details and results are given in the next sections.

### 3.2. Rapid Quality Diagnosis (QD)

The goal of the product-quality-monitoring QD tool is to be able to diagnose the manufacturing process conditions, manufactured product quality, and potential failure causes quickly and effectively. In particular, by using ML techniques on industrial sensor signals, it applies intelligent techniques to enhance the quality of the finished product. It also performs sophisticated statistical analysis (e.g., causality analysis) on the manufacturing conditions and machine parameters to determine the factors that have the greatest impacts on the product quality. This process is essential because industrial data typically exhibit complex relationships and unpredictable dynamics, the exact signal evolution patterns are not very deep, and latent factors that impact the manufacturing process as a whole are difficult for human operators to directly identify. For the purpose of optimally reconfiguring the machine parameters and implementing corrective measures to mitigate potential product defects, the analysis results are sent to alternative solutions or directly to the machine operators. When a defective product is found, the product-quality-monitoring tool generates an output that includes the relevant parameter configurations of the observations where the failure is thought to be present, along with an alert. Ultimately, it offers assessment visuals to help the machine operators monitor the entire analysis process closely. Three main objectives need to be met in order to achieve zero-defect manufacturing: (i) the cutting of costs and waste, (ii) the elimination of defects, and (iii) the enhancement of the overall quality of the production.

The primary features of this solution are quick product quality diagnosis and intelligent alerting upon the detection of a defective product. This is a microservice that guarantees extremely precise product quality monitoring throughout the manufacturing process. Additionally, it offers crucial parameter configurations that could lead to product malfunctions and offers suggestions for quick reconfigurations. It finds flaws in the quality of the product and generates alerts to notify a human operator, or finds other analytical solutions in a timely manner, allowing the necessary corrective actions to be carried out before the product fails permanently.

**Figure 2:**
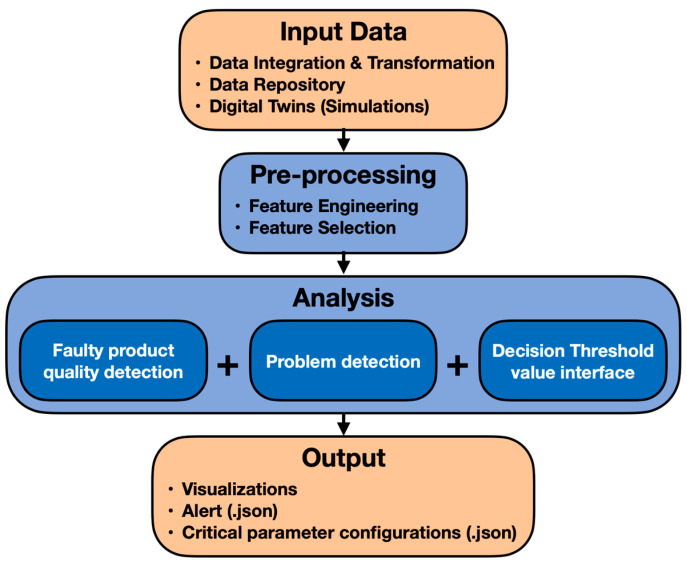
Conceptual architecture of product-quality-monitoring QD tool.

A diagram of the QD conceptual framework is shown in Figure 2.

#### QD Framework for Chatter Detection

The QD tool has been effectively employed for identifying chatter in CNC machining processes. Similar steps to those of the IM design process were taken to guarantee the complete functionality of the chatter detection framework, with a focus on its ability to generalize:

1. Employ a survey study to pick a set of robust classifiers that have previously been utilized for chatter detection;
2. Apply feature engineering techniques on a real industrial chatter dataset;
3. Choose the most effective classifier from the experiments;
4. Optimize the top-performing algorithm to ensure a high level of detection accuracy;
5. Finalize the QD tool, including the alerting mechanism, critical CNC parameter configurations, and visualizations.

The goal is to determine the presence or absence of chatter in the input observations. This task involves binary classification, and, as a result, binary classifiers were employed during the implementation phase. After multiple experiments, the LGBM was selected based on its classification performance and scalability compared to the other classifiers (SVM, RF, and XGBOOST).

## 4. Evaluation

### 4.1. Quantitative Results

#### 4.1.1. IM: Experiments and Results

The evaluation was performed on a specific component degradation dataset from Fidia [61]. It was divided into two different folders, with the degraded-component data in the first case and the normal ones in the other case. A total of 67 JSON files (each one corresponding to a different experiment) include information about the degraded-component observations, while 37 JSON files include information about the normal ones. The respective labels were constructed based on this categorization. Specifically, the component degradation Boolean variable was introduced, which is equal to “1” in the degradation presence case and to “0” if otherwise. The features (independent variables) included in this dataset are as follows:

1. XVibration: vibration of X-axis (mm/s^2^);
2. YVibration: vibration of Y-axis (mm/s^2^);
3. ZVibration: vibration of Z-axis (mm/s^2^);
4. XPosition: position of X-axis (mm);
5. YPosition: position of Y-axis (mm);
6. ZPosition: position of Z-axis (mm);
7. XMotorCurrent: motor current of X-axis (A);
8. YMotorCurrent: motor current of Y-axis (A);
9. ZMotorCurrent: motor current of Z-axis (A).

The data are organized into chunks with information about the oldest, currently supplied, and last available chunks. Extra information regarding the sampling techniques, timestamps, etc. are also provided within each JSON file. The contributions of each feature are gathered in Table 1.

The percentages presented in the following tables refer to the individual contribution of each feature to accurately detecting the presence of component degradation and machine chatter of IM and QD, respectively. The binary classification accuracy was calculated based on the following mathematical formula:

$$Accuracy=\frac{TP+TN}{TP+TN+FP+FN}$$

The respective feature importance values are gathered in Table 2. These values refer to the involvement that each feature had in the predictions of the model. Specifically, the importance countifies the improvement in the accuracy or purity achieved by a split for each decision tree classifier of which the LGBM is comprised. It is a measure of the quality of a split based on a particular feature.

As can be seen, the most important feature, enhancing the component degradation detection performance of the LGBM, is the median value of the X-axis position, while the FFT of the Y-axis position does not contribute at all to the performance. An interesting fact is that, in the XGBoost, the feature importance values are significantly different. Specifically, the most important feature enhancing the tool wear/component degradation performance is the motor current of the Z-axis.

The LGBM algorithm was fine-tuned in order to ensure its efficacy. The most significant parameters that were fine-tuned were (1) the learning rate, which determines the step size at each iteration while moving towards the minimum of a binary log loss function, and (2) the number of estimators, which defines the number of gradient-boosted trees, equivalent to the number of boosting rounds. The optimal parameters were computed by performing a grid search, using 10-fold cross-validation for the aforementioned model parameters, evaluating a series of values: (1) the number of estimators = (5, 10, 20, 50, 100), (2) the learning rate = (0.001, 0.01, 0.02, 0.3). The maximum value for the learning rate was set to 0.3 in order to ensure a detailed search and avoid skipping a possible minimum. The results are summarized in Table 3.

As discussed in the previous section, in addition to the LGBM, the RF, SVM, and XGBoost classifiers were also evaluated in Fidia’s dataset. Table 4 gathers the experimental results.

The LGBM performed the best. This component degradation framework was also successfully and easily applied on a public CNC tool wear dataset in Kaggle1, reaching a test accuracy equal to 96.74%. This fact indicates that the generalization goal of the solution was reached.

#### 4.1.2. QD: Experiments and Results

First, feature engineering and pre-processing were applied on Fidia’s chatter dataset. This CNC chatter dataset comprises 180,224 observations, with the fast Fourier transform (FFT) calculated for the vibrations of the X, Y, and Z axes. The training set was created using a five-fold cross-validation approach, encompassing 80% of the initial dataset, which included 154,478 samples. The remaining 20% of the initial dataset was designated as the test set, containing 25,746 samples.

Moreover, apart from computing the average value of each feature entry, the corresponding median value was also computed to introduce an additional feature, thereby enhancing the performance of the LGBM. Additionally, the FFT of the position of the Y-axis was introduced to improve the chatter detection performance. The dataset is organized into two distinct folders: one containing observations with chatter and the other containing normal observations. In the first case, there are 11 JSON files providing information about the observations with chatter, while the second case includes 16 JSON files with information about the normal observations. Each JSON file corresponds to a unique experiment.

To label the data, a Boolean variable representing chatter presence is assigned to each observation. It is set to “1” for instances in which chatter is present and to “0” for instances in which chatter is absent. The features of the observations are detailed as follows:

1. XVibration: vibration of X-axis (mm/s^2^);
2. YVibration: vibration of Y-axis (mm/s^2^);
3. ZVibration: vibration of Z-axis (mm/s^2^);
4. XPosition: position of X-axis (mm);
5. YPosition: position of Y-axis (mm);
6. ZPosition: position of Z-axis (mm);
7. XMotorCurrent: motor current of X-axis (A);
8. YMotorCurrent: motor current of Y-axis (A);
9. ZMotorCurrent: motor current of Z-axis (A).

The contribution of each feature to the detection performance of the algorithm is presented in Table 5.

The respective feature importance values are gathered in Table 6.

Clearly, the most important feature that enhances the chatter detection performance of the LGBM is the Butterworth filtering of the vibrations of the X-axis. To find the optimal parameter setting of the LGBM, a grid search was utilized. The results are gathered in Table 7.

Similar to Section 4.1.1, a performance comparison between the RF, SVM, XGBoost, and LGBM classifiers was conducted to select the best-performing one. As can be seen in Table 8, the LGBM performed the best once again.

### 4.2. Qualitative Results

#### 4.2.1. IM: Visualizations of Results

**Figure 3:**
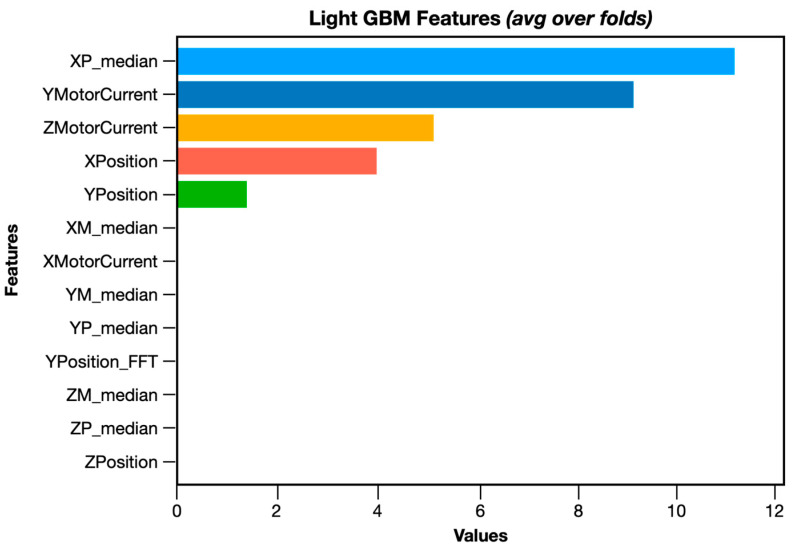
LGBM feature importance visualization.

Some visualizations of the qualitative results regarding the component degradation detection framework applied on Fidia’s use case are presented below. Figure 3 illustrates the feature importance used in the LGBM. As can be seen, the most important feature contributing to the detection performance is the median value of the *X*-axis position.

**Figure 4:**
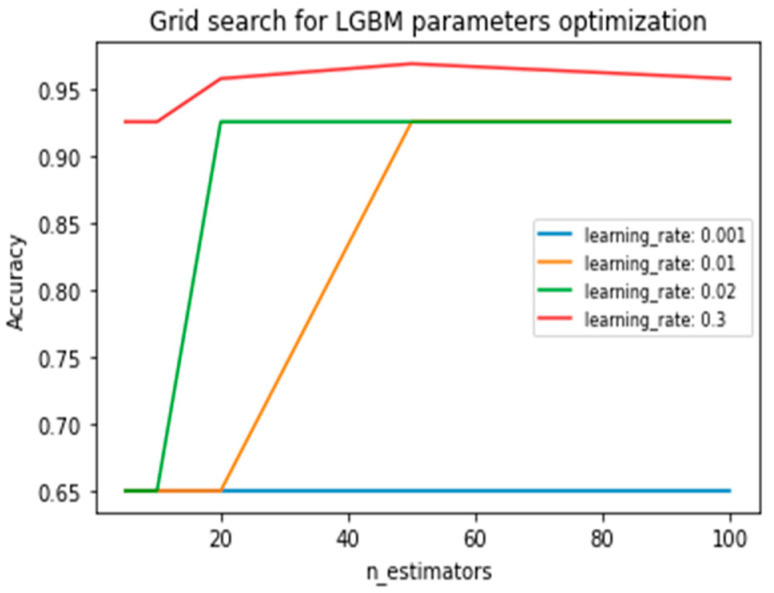
LGBM parameter setting vs. tool wear detection accuracy.

Figure 4 presents a visualization of the grid search conducted to optimize the parameter configuration of the LGBM.

#### 4.2.2. QD: Visualizations of Results

**Figure 5:**
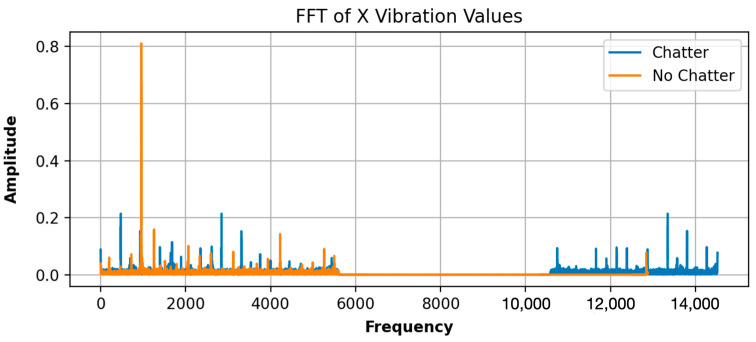
Computed FFT X-axis vibration values of observation with chatter presence and absence, respectively.

To gain a more comprehensive insight into the analysis results, visualizations of the variables that exert the most significant influence on chatter detection are provided. In the case of machine vibrations, which are the variables under evaluation for Fidia, FFT is employed to convert motion data, over time, yielding an understandable output that quantifies the extent of the recorded vibrations, as depicted in Figure 5. The amplitude signifies the intensity of the vibrations present, while the frequency indicates the number of movements in hertz (Hz).

**Figure 6:**
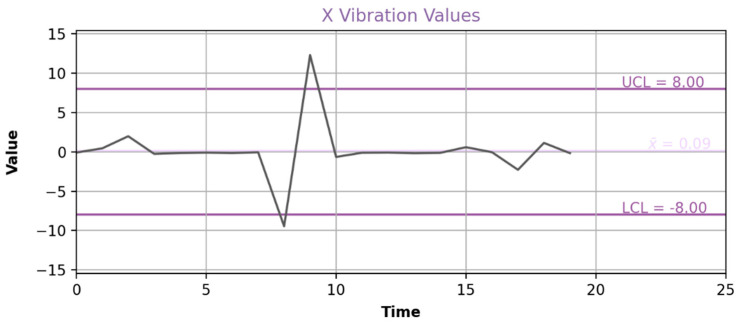
Real-time visualization of the X-axis vibrations.

Apart from the FFT visualization (Figure 5), there is an option to represent machine vibrations on the fly, offering the potential for real-time decision support. Consequently, the vibration parameters are organized as time-series data. As illustrated in Figure 6, the mean of the displayed sample is presented, and the Upper and Lower Critical Limits (UCL, LCL) can be incorporated to define the desired quality range. It is important to note that this visualization relies on specialized technologies for handling data streams, necessitating a pre-established connection to the relevant data stream.

**Figure 7:**
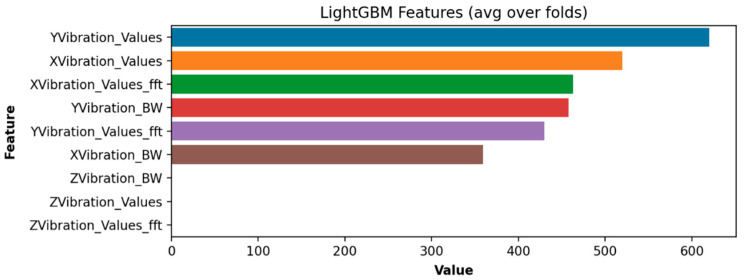
Feature importance visualization.

**Figure 8:**
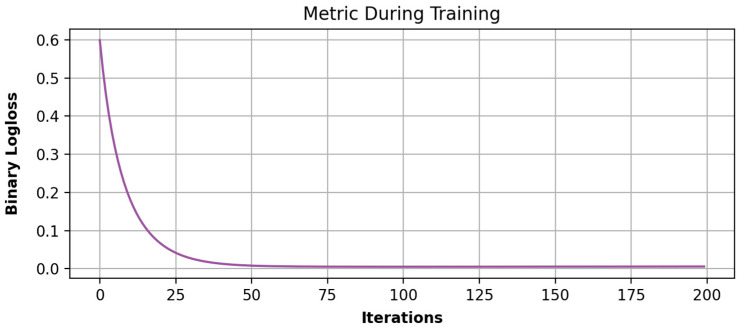
LGBM binary log loss during training iterations.

Some visualizations of the most important features of the chatter detection framework and the binary log loss values obtained during 200 training iterations are presented in Figure 7 and Figure 8, respectively.

## 5. Discussion and Conclusions

Industry 4.0 has set standards and requirements, which smart industries are called to follow. In order to meet these requirements, smart CNC machining requires high efficiency and automated systems with the minimum or—if possible—no human interaction with the machines. Therefore, CNC machines have started using advanced control technologies, such as artificial intelligence (AI) systems and machine-learning mechanisms. Although having a specialized CNC machine is expensive for an industry, it offers many benefits and is necessary in order to achieve high-precision machining: it allows for much more control over the machine, a larger exploitation of its manufacturing capabilities, and uncanny access to the data.

With respect to the quality in CNC machining, the two most important characteristics are the dimensional accuracy and the surface roughness, which are usually evaluated off-line by adequate measurements of the processed pieces. Machine vibrations, corresponding to the relative movement between the workpiece and the cutting tool, pose a serious threat to this quality, as they affect the smoothness of the workpiece surface, reduce its accuracy, produce harsh noise, and accelerate the tool wear, among other undesired consequences. To address these formidable challenges and maintain a competitive edge in the CNC machining landscape, companies like Fidia are engaged in a perpetual quest for innovation. Their commitment to excellence extends beyond precision machining; it encompasses a holistic approach to optimizing every facet of the manufacturing process.

The recent advancements in CNC tool wear prediction, chatter detection, and surface roughness prediction present notable research gaps, particularly concerning the generalization ability of certain models to real-life CNC machining processes. Models like ANNs and decision tree-based classifiers exhibit limitations in industrial settings, as they are typically trained within specific parameter ranges and conditions, leading to decreased monitoring accuracy when the conditions change and labeled data may not be readily available. Moreover, while SVMs have been widely applied, their performance is highly dependent on the parameter settings, and the computational complexity of optimizing these parameters.

The integration of the tools that are presented in this article represents a pivotal milestone in the pursuit of CNC machining excellence, aiming to tackle the generalization problem though the adoption of a machine- and configuration-agnostic approach. This approach ensures accurate predictions by relying solely on data derived from vibration sensors, which are ubiquitous in CNC machines. The algorithm’s performance is further optimized through the implementation of feature extraction techniques, enhancing the existing vibration data and contributing to more robust and accurate predictions across various CNC machine setups and configurations. This standardized and sensor-centric methodology improves the solutions’ adaptability to diverse machining conditions, mitigating the limitations associated with specific parameter ranges and conditions. As a result, the IM and QD tools have been meticulously designed to effectively address the multifaceted challenges encountered in high-speed machining processes related to vibrations. Specifically, IM has been successfully applied for tool wear/component degradation detection, while QD has been utilized for chatter detection, both achieving correct problem prediction accuracies of over 95% in their corresponding domains. These tools serve as the linchpins in addressing the machine vibrations and chatter problems that have been meticulously analyzed using machine learning. By continuously monitoring the machining environment and processing vast amounts of data, these tools can not only identify the sources of vibration but also proactively mitigate them.

The result is a dramatic reduction in the adverse effects of vibrations, including improved surface quality, enhanced dimensional accuracy, reduced tool wear, and a quieter machining environment. These advanced technologies provide not just solutions to existing challenges but also new horizons for optimizing production processes. The synergy between precision engineering and data-driven intelligence is set to redefine CNC machining, setting new benchmarks for efficiency, quality, and innovation.

In light of the promising outcomes presented in this scientific paper, the future scope of our research entails broadening the applicability of the proposed solution beyond its current focus on metal machining. The next steps involve exploring and adapting the methodology to diverse manufacturing industries, such as plastic injection molding and wood machining. By extending the reach of our proposed solution to these domains, we aim to evaluate its effectiveness in addressing unique challenges and optimizing processes across a spectrum of materials and manufacturing techniques. This expansion not only contributes to the versatility of the proposed approach but also enhances its potential impact on industrial practices, paving the way for more comprehensive and inclusive advancements in the field.

## Figures and Tables

**Table 1:** Feature contributions to prediction accuracy.

Features Used	LGBM Detection Accuracy
XPosition	60%
YPosition	65%
ZPosition	65%
XMotorCurrent	65%
YMotorCurrent	80.2%
ZMotorCurrent	82.5%
XP_median	95.27%
YP_median	95.27%
ZP_median	95.27%
XM_median	95.27%
YM_median	95.27%
ZM_median	95.27%
YPosition_FFT	95.27%

**Table 2:** Feature importance values (LGBM).

Feature Used	Importance Value
XP_median	11
YMotorCurrent	9
ZMotorCurrent	5
XPosition	4
YPosition	1
ZPosition	0
XMotorCurrent	0
YP_median	0
ZP_median	0
YPosition_FFT	0

**Table 3:** LGBM grid search results.

Features Used		LGBM Detection Accuracy
‘learning_rate’: 0.001	‘n_estimators’: 5	65%
‘learning_rate’: 0.001	‘n_estimators’: 10	65%
‘learning_rate’: 0.001	‘n_estimators’: 20	65%
‘learning_rate’: 0.001	‘n_estimators’: 50	65%
‘learning_rate’: 0.001	‘n_estimators’: 100	65%
‘learning_rate’: 0.01	‘n_estimators’: 5	65%
‘learning_rate’: 0.01	‘n_estimators’: 10	65%
‘learning_rate’: 0.01	‘n_estimators’: 20	65%
‘learning_rate’: 0.01	‘n_estimators’: 50	92.56%
‘learning_rate’: 0.01	‘n_estimators’: 100	92.56%
‘learning_rate’: 0.02	‘n_estimators’: 5	65%
‘learning_rate’: 0.02	‘n_estimators’: 10	65%
‘learning_rate’: 0.02	‘n_estimators’: 20	92.56%
‘learning_rate’: 0.02	‘n_estimators’: 50	92.56%
‘learning_rate’: 0.02	‘n_estimators’: 100	92.56%
‘learning_rate’: 0.3	‘n_estimators’: 5	92.56%
‘learning_rate’: 0.3	‘n_estimators’: 10	92.56%
‘learning_rate’: 0.3	‘n_estimators’: 20	95.76%
‘learning_rate’: 0.3	‘n_estimators’: 50	96.89%
‘learning_rate’: 0.3	‘n_estimators’: 100	95.76%

**Table 4:** Performance comparison between RF, SVM, XGBoost, and LGBM.

Classifier	Accuracy
RF	91.3%
SVM	72.09%
XGBoost	91.27%
LGBM	96.89%

**Table 5:** Feature contributions to chatter detection accuracy.

Feature Used	LGBM Detection Accuracy
XVibration_Values	61.2%.
YVibration_Values	68.5%
ZVibration_Values	68.5%
XVibration_Values_fft	65%
YVibration_Values_fft	80.2%
ZVibration_Values_fft	82.5%
XVibration_BW	95.27%
YVibration_BW	95.27%
ZVibration_BW	95.27%

**Table 6:** QD feature importance values (LGBM).

Feature Used	Importance Value
XVibration_BW	1144
YVibration_Values_fft	1029
YVibration_BW	958
XVibration_Values_fft	915
YVibration_Values	899
YVibration_Values	762
ZVibration_BW	0
ZVibration_Values	0
ZVibration_Values_fft	0

**Table 7:** QD LGBM grid search results.

Features Used		LGBM Detection Accuracy
‘learning_rate’: 0.001	‘n_estimators’: 5	91.75%
‘learning_rate’: 0.001	‘n_estimators’: 10	91.76%
‘learning_rate’: 0.001	‘n_estimators’: 20	91.77%
‘learning_rate’: 0.001	‘n_estimators’: 50	91.64%
‘learning_rate’: 0.001	‘n_estimators’: 100	91.72%
‘learning_rate’: 0.01	‘n_estimators’: 5	91.63%
‘learning_rate’: 0.01	‘n_estimators’: 10	91.7%
‘learning_rate’: 0.01	‘n_estimators’: 20	91.78%
‘learning_rate’: 0.01	‘n_estimators’: 50	92.82%
‘learning_rate’: 0.01	‘n_estimators’: 100	91.72%
‘learning_rate’: 0.02	‘n_estimators’: 5	91.74%
‘learning_rate’: 0.02	‘n_estimators’: 10	92.05%
‘learning_rate’: 0.02	‘n_estimators’: 20	92.82%
‘learning_rate’: 0.02	‘n_estimators’: 50	93.45%
‘learning_rate’: 0.02	‘n_estimators’: 100	93.44%
‘learning_rate’: 0.3	‘n_estimators’: 5	93.17%
‘learning_rate’: 0.3	‘n_estimators’: 10	94.05%
‘learning_rate’: 0.3	‘n_estimators’: 20	95.17%
‘learning_rate’: 0.3	‘n_estimators’: 50	95.8%
‘learning_rate’: 0.3	‘n_estimators’: 100	95.98%

**Table 8:** Performance comparison between RF, SVM, XGBoost, and LGBM for chatter detection.

Classifier	Accuracy
RF	82.93%
SVM	60%
XGBoost	90.47%
LGBM	95.98%

## Data Availability

Data are unavailable due to privacy restrictions.
